# Interstitial Lung Diseases Misdiagnosed as Tuberculosis

**DOI:** 10.12669/pjms.342.14407

**Published:** 2018

**Authors:** Nousheen Akhter, Nadeem Ahmed Rizvi

**Affiliations:** 1 Dr. Nousheen Akhter, FCPS Trainee. Department of Chest Medicine, Jinnah Postgraduate Medical Centre, Karachi, Pakistan; 2 Prof. Nadeem Ahmed Rizvi, FRCP. Department of Chest Medicine, Jinnah Postgraduate Medical Centre, Karachi, Pakistan

**Keywords:** Antitubercular agents, Mycobacterium infection, Interstitial lung diseases

## Abstract

**Objective:**

To determine the frequency of misdiagnosis of tuberculosis in interstitial lung disease cases.

**Methods:**

This is a prospective study including patients registered in the interstitial lung disease clinic, Jinnah Postgraduate Medical Center, Karachi, during May–June 2017. Diagnosis of tuberculosis was only confirmed if there was any bacteriological evidence of tuberculosis at the time of diagnosis or if there was improvement in symptoms after treatment in patients diagnosed as having tuberculosis on clinical grounds.

**Results:**

Seventy-three patients were included in the study, out of which 53 (72.60%) were females and 20 (27.39%) were males. Tuberculosis was treated before presentation in 28 (38.35%) of interstitial lung disease patients. Except for two silicosis patients who had smear positive tuberculosis, rest of the patients were misdiagnosed as having tuberculosis.

**Conclusion:**

Interstitial lung diseases are the disorders that are frequently unrecognized and misdiagnosed. More commonly the confusion is with tuberculosis. Thorough knowledge about interstitial lung diseases should be provided to the primary care physicians, especially in countries with high tuberculosis burden, so that to limit maltreatment with anti-tuberculous drugs when they are not needed and early referral to interstitial lung disease clinic.

## INTRODUCTION

Interstitial lung diseases (ILDs) are a heterogeneous group of diseases that affect the lung parenchyma.^1^ They have variable aetiologies, signs and symptoms, radiographic and histologic features. Approximately two hundred entities are included in this group of diseases, the most common being idiopathic pulmonary fibrosis (IPF).^2^

There is a paucity of data on ILD in Pakistan^3^, where these diseases are under-estimated and remain under-diagnosed and under-reported for various reasons. This is probably due to lack of awareness among physicians, and the high cost of diagnostic modalities. Dynamic interactions between clinicians, radiologists, and pathologists improve inter-observer agreement and diagnostic confidence.^4^

Most of the fibrotic ILDs carry a dismal prognosis with a median survival time for IPF of two to three years from the time of diagnosis.^5^-^8^ The diagnosis is not straightforward in most of the cases; Not surprisingly, >50% of these patients are initially misdiagnosed with other forms of respiratory illness.^9^,^10^ Timely diagnosis and early referral of patients to centres with specific expertise may lead to more optimal disease management.

Mycobacterium tuberculosis (MTB) is a relatively common cause of chronic lung infections worldwide.^11^ In studies conducted in the 1970-90s that examined patients with idiopathic chronic interstitial lung disease, the positive culture rate for MTB was 5%-6.2%.^12^,^13^ This incidence was 4-5 times higher than that of the general population in the same country at the same time.

Tuberculosis mimics the clinical and radiological features of many ILDs, leading to diagnostic errors and delays.^14^ Due to decreased awareness of primary care physicians about ILDs; they are frequently misdiagnosed as TB. There are only a few case reports from African countries where ILDs, especially IPF were initially wrongly diagnosed and managed as tuberculosis.^15^-^18^

Pakistan ranks 5^th^ among 22 countries with the highest burden of TB epidemic.^19^ Total population of the country are 193 million and the incidence of tuberculosis is reported to be 268/100,000. Mortality from tuberculosis is reported to be 23/100,000 population.^20^

To the best of our knowledge, no data exist on this subject. Therefore, this study was conducted to investigate the frequency of interstitial lung diseases which were misdiagnosed as tuberculosis.

## METHODS

This is a prospective study which included all consecutive patients visiting the interstitial lung disease clinic, Jinnah Postgraduate Medical Center, Karachi, during May–June 2017. Interstitial lung disease clinic is a specialized clinic at department of chest medicine, Jinnah postgraduate medical center, which runs three days a week and around ten patients visit the clinic per day.

Diagnosis of interstitial lung disease was made on detailed medical history, clinical examination, HRCT chest findings consistent with interstitial lung diseases, serological tests and response to immunosuppressive treatment. In those patients where there was possibility of diseases other than ILD bronchoscopy with lavage ± biopsy was done.

The diagnosis of TB was only confirmed if there was a record of positive smear or any bacteriological evidence when TB was diagnosed. For patients who had been treated for smear negative tuberculosis, the diagnosis of tuberculosis was refuted if the medical history and radiological features were inconsistent with tuberculosis and they failed to respond to anti-tuberculous medicines.

### Statistical analysis

Statistical package for the Social Sciences (IBM SPSS, United States), version 23 was used for statistical analysis. Frequencies and percentages were calculated for quantitative variables. This study was approved by the hospital’s ethical committee. Written informed consent was obtained from each patient.

## RESULTS

Seventy-three patients were included in the study, out of which 53 (72.60%) were females and 20 (27.39%) were males. Mean age of the study population was 48.92 years (range: 17 – 80 years). ILDs were treated as tuberculosis before presentation to the ILD clinic in 28 (38.35%) patients. The frequency of misdiagnosis of tuberculosis in common interstitial lung diseases is shown in Table-I.

**Table-I:** Frequency of interstitial lung diseases treated as tuberculosis before presentation to ILD clinic.

ILD	Number of cases presented to ILD clinic	Cases treated as TB before presenting to ILD clinic	

		Frequency	Percentage
IPF	32	21	65.62
NSIP	26	2	7.69
Sarcoidosis	5	1	20
Silicosis	3	3*	100
Hypersensitivity pneumonitis	7	1	14.28

ILD: Interstitial lung disease, IPF: Idiopathic pulmonary fibrosis, NSIP: Non-specific interstitial pneumonia.

* 2 out of 3 silicosis patients had smear positive tuberculosis in the past. Rest of the interstitial lung disease patients were misdiagnosed as having tuberculosis and were wrongly treated with anti-tuberculous drugs.

Among all the ILD patients, only 2 patients who were later diagnosed as having silicosis had sputum smear positive tuberculosis in past and were treated for it. Rest of them were misdiagnosed initially as having tuberculosis and were given anti-tuberculous treatment but had no improvement in their symptoms.

## DISCUSSION

Tuberculosis is frequently considered by primary care physicians to be the cause of symptoms of interstitial lung disease patients. As shown in the present study, 38.35% of patients having chronic interstitial lung disease were managed in the past as having sputum smear negative tuberculosis, even though there was no improvement in symptoms of the patient subjectively or objectively.

Only two patients were correctly treated as having TB, both were diagnosed later as also having silicosis. Both patients had sputum smear results which were positive for TB. The elevated risk of tuberculosis in patients with silicosis is widely known. Several researchers have reported this in their work throughout the globe.^21^,^22^

The prevalence of tuberculosis is high in IPF.^23^ This is because of the impairment of immunity caused by diffuse structural changes due to fibrosis. And, also due to the use of corticosteroids, the immune system is weakened causing latent TB to become active. In our study, none of the patients with IPF had any bacteriological or clinical evidence of having tuberculosis in the past. Their symptoms of chronic dyspnoea, dry cough and generalized ill health were wrongly attributed to as due to TB.

A great degree of clinical suspicion is required to diagnose ILDs earlier. In fact, the most common symptoms of cough and dyspnoea tend to be overlooked and wrongly thought to be due to smoking habits, aging or some infections, more commonly tuberculosis. This is in part due to the decreased awareness of primary care physicians about various interstitial lung diseases and due to lack of resources in the region to get diagnostic tests like computed tomography scan and lung biopsy. This leads to many patients being subjected to unnecessary anti-tuberculous drugs and their side effects and causes a delay in accessing appropriate care.

The delay in diagnosis of such fibrotic diseases, especially IPF, with little median survival is consequential. This leads to higher mortality independent of disease severity.^24^

## CONCLUSION

Interstitial lung diseases are the disorders that are frequently unrecognized and misdiagnosed. More commonly the confusion is with tuberculosis. Thorough knowledge about interstitial lung diseases should be provided to the primary care physicians, especially in countries with high tuberculosis burden, so that to limit maltreatment with anti-tuberculous drugs when they are not needed and early referral to interstitial lung disease clinic.

### Author`s contribution

**NAR** conceived, designed and reviewed the manuscript.

**NA** did data collection, statistical analysis and manuscript writing.
